# Evaluation of the ICT Tuberculosis test for the routine diagnosis of tuberculosis

**DOI:** 10.1186/1471-2334-6-37

**Published:** 2006-02-27

**Authors:** Gozde Ongut, Dilara Ogunc, Filiz Gunseren, Candan Ogus, Levent Donmez, Dilek Colak, Meral Gultekin

**Affiliations:** 1 Department of Medical Microbiology, Akdeniz University, Faculty of Medicine, Antalya, Turkey; 2 Department of Infectious Diseases and Clinical Microbiology, Akdeniz University, Faculty of Medicine, Antalya, Turkey; 3 Department of Respiratory Medicine, Akdeniz University, Faculty of Medicine, Antalya, Turkey; 4 Department of Public Health, Akdeniz University, Faculty of Medicine, Antalya, Turkey

## Abstract

**Background:**

Rapid and accurate diagnosis of tuberculosis (TB) is crucial to facilitate early treatment of infectious cases and thus to reduce its spread. To improve the diagnosis of TB, more rapid diagnostic techniques such as antibody detection methods including enzyme-linked immunosorbent assay (ELISA)-based serological tests and immunochromatographic methods were developed. This study was designed to evaluate the validity of an immunochromatographic assay, ICT Tuberculosis test for the serologic diagnosis of TB in Antalya, Turkey.

**Methods:**

Sera from 72 patients with active pulmonary (53 smear-positive and 19 smear-negative cases) and eight extrapulmonary (6 smear-positive and 2 smear-negative cases) TB, and 54 controls from different outpatient clinics with similar demographic characteristics as patients were tested by ICT Tuberculosis test.

**Results:**

The sensitivity, specificity, and negative predictive value of the ICT Tuberculosis test for pulmonary TB were 33.3%, 100%, and 52.9%, respectively. Smear-positive pulmonary TB patients showed a higher positivity rate for antibodies than smear-negative patients, but the difference was not statistically significant. Of the eight patients with extrapulmonary TB, antibody was detected in four patients.

**Conclusion:**

Our results suggest that ICT Tuberculosis test can be used to aid TB diagnosis in smear-positive patients until the culture results are available.

## Background

A curable and preventable disease, tuberculosis (TB) continues to be a leading cause of mortality and morbidity worldwide. Early treatment of infectious cases reduces spread of TB. Therefore rapid and accurate identification of infected individuals is mandatory [1,2].

Currently, mycobacteriology laboratory algorithm to detect *Mycobacterium tuberculosis *(*M. tuberculosis*) consists of two steps: microscopic examination of a smear prepared from a concentrated specimen (sputum, bronchoalveolar lavage fluid, aspirates, etc) and culture. Smear microscopy allows direct detection of acid fast bacilli (AFB) in the specimen and identification of the most contagious patients. Although smear microscopy provides rapid results and inexpensive ways to diagnose TB, it has limitations. Probably the most important limitation is low sensitivity. Behr et al. reported that smear examination can detect less than 50% of all culture positive patients [3]. Culture, on the other hand, is more effective than smear microscopy. While 10^4 ^AFB/ml of specimen usually result in 60% of smear positivity, only ten viable bacilli per milliliter are required for culture positivity. The sensitivity of culture is 80%–85%. Additionally, identification and susceptibility testing of the isolates are major advantages of the cultural methods except that they take relatively long growth time. Today, TB diagnosis in resource limited countries mostly depends on clinical and radiological findings, as well as sputum smear microscopy and culture [4,5].

In response to the need for a rapid diagnosis of TB, a number of new approaches and methods were developed for the serological diagnosis. There are several serological tests, which use various native or recombinant antigens such as 38-kDa antigen, lypoarabinomannan, antigen 60 (A60), and tuberculous glycolipids (TBGL's). These include several enzyme-linked immunosorbent assay (ELISA)-based serological tests and immunochromatographic methods to detect antibodies to *M. tuberculosis *[6-10].

According to WHO Report 2002 on Global Tuberculosis Control, Turkey was classified in Category 1, which includes countries not implementing the DOTS strategy and having an estimated incidence rate of 10 or more cases per 100 000 population. In this report, a number of 18 038 of diagnosed cases and an incidence rate of 27 per 100 000 population of TB for Turkey were reported [11]. However this data do not reflect the true incidence of TB in Turkey due to underreporting and undiagnosed cases [12].

In this study, we evaluated an immunochromatographic assay, ICT Tuberculosis test which detects serum antibodies against five antigens that are secreted by *M. tuberculosis *during active infection by determining its sensitivity and specificity as compared to standard diagnostic procedures in a university hospital setting in Turkey.

## Methods

### Patients

Between April 1999 and December 2000, 80 patients with active TB were evaluated. All were human immunodeficiency virus negative. There were 15 female (18.75%) and 65 males (81.25%) aged between 14 to 76 years (median 39 years). Of the 80 patients with active TB, 72 (90%) had pulmonary disease and eight (10%) had extrapulmonary disease. Among patients with pulmonary disease, 53 (73.6%) patients were both smear and culture positive, 19 (26.4%) patients were smear-negative and culture-positive. Extrapulmonary disease included pleural disease (three patients), lymphadenitis (four patients), and epididymitis (one patient). Of the eight patients with extrapulmonary disease, two were both smear and culture positive, six patients were only culture-positive.

### Control group

The control group consisted of 54 individuals selected randomly from individuals who applied to different outpatient chest clinics for employment TB screening. All members of control group had no previous history of TB, no signs or symptoms suggestive of pulmonary TB, no evidence of TB on chest X rays.

The 54 subjects selected for the control group had a median age of 41 years (age range, 18–75 years) and 43 (79.6%) were males, 11 (20.4%) were females. All were HIV negative. Demographic characteristics of patients and control subjects were similar.

Serum samples were obtained from almost all patients before initiation of antituberculosis treatment and stored at -80°C until tested. All patients and controls participated in the study were vaccinated with *Mycobacterium bovis *BCG.

Akdeniz University Medical Faculty Ethical Committee approved this study.

### Microbiological analysis

Routine TB examination included demonstration of AFB in Ziehl-Neelsen stained smears from sputum, bronchoalveolar lavage (BAL) fluid, aspirates or tissue biopsy samples, and culture according to standard procedures [5].

### ICT Tuberculosis test

The ICT Tuberculosis diagnostic kit (ICT Diagnostics, Bangowlah, New South Wales, Australia) is designed for the detection of antibodies to *M. tuberculosis*. Briefly, five highly purified antigens (including one of 38 kDa) secreted by *M. tuberculosis *during active infection are immobilized in four lines on the test strip. When serum or plasma applied, it flows past the antigen line. Bound antibody is detected by a goat anti-human IgG antibody conjugated to colloidal gold particles which produces one or more pink lines when bound to human antibody. The whole procedure is completed within 20 minutes. ICT Tuberculosis test doesn't require special equipment and technical skill.

### Statistical analysis

Validity of ICT Tuberculosis test was measured by sensitivity and specificity. Negative predicitive value was also calculated. Antibody positivity rates between smear-positive and smear-negative patients were compared by chi-square test.

## Results

Antibody was detected in 21 of 53 (29.2%) smear and culture-positive patients, three of 19 (4.2%) smear-negative culture-positive patients. Patients with smear positivity showed a higher positivity rate for antibodies than smear-negative patients but the difference was not statistically significant (χ^2^: 3.57, p: 0.058). We found the sensitivity 33.3% and specificity 100% with a negative predictive value of 52.9% for pulmonary TB.

Of the eight patients with culture-proven extrapulmonary TB, antibody was detected in four patients. Sensitivity of the test for the extrapulmonary TB patients was not calculated because of the small number of the patients. None of the control subjects tested positive by the ICT Tuberculosis test.

## Discussion

Serologic tests are the oldest methods for the diagnosis of TB. Agglutination of patient's serum with *M. tuberculosis *was investigated first in 1898 [13]. Since then, a number of serologic tests were developed for detection of host response to *M. tuberculosis*, but none of them has found widespread clinical use. The specificity of the early serological tests which were prepared from crude antigens were low because of cross reaction with environmental Mycobacterium species. Specificity could be improved by using purified antigens. A variety of antigens have been adapted for the serodiagnosis of TB. Certain antigens were found to have more diagnostic significance, one of them, 38 kDa antigen which is a phosphate-binding protein, was reported to be specific to the *M. tuberculosis *complex. This antigen has been identified as a potential reagent to be used for the screening of TB [5].

Cole et al. [14] evaluated a rapid membran-based antibody assay which contained only the 38 kDa antigen, for the diagnosis of active pulmonary TB in China and reported a sensitivity of 89% for smear-positive patients and a sensitivity of 74% for smear-negative patients, with a specificity of 93%. Zhou et al. [15] have expanded the control and patient groups and added an extrapulmonary TB group in that they found similar results.

In our study, we evaluated the ICT Tuberculosis test to detect IgG antibodies to five antigens (one of them was p38) which were secreted by *M. tuberculosis *in patients with TB and found that sensitivity and specificity were 33.3%, 100%, and negative predictive value was 52.9%, respectively, for pulmonary TB.

Previous investigators reported that the sensitivity of ICT Tuberculosis test for pulmonary TB is variable, ranging from 20% to 73%, specificity rates were reported as 80–100% [7,16-23]. We found a sensitivity of 33.3% for ICT Tuberculosis test with values ranging from 39.6% to 15.8% in smear-positive and smear-negative patients, respectively. As in previous studies, we found that sensitivity of ICT Tuberculosis test is higher in smear-positive patients than smear-negative patients, but the difference was not statistically significant [7,14,15,17,19,22]. It is most likely that reported higher sensitivity in smear-positive patients may be due to the higher bacillary loads and thus a greater exposure to antigens and a more vigorous antibody response in these patients.

## Conclusion

In conclusion, ICT Tuberculosis test can be used to aid TB diagnosis in smear-positive patients until the culture results are available.

## Competing interests

The author(s) declare that they have no competing interests.

## Authors' contributions

DO conceived and designed the study, conducted the laboratory studies and involved in writing of the manuscript. GO participated in the study design, carried out the laboratory studies and helped in writing of the manuscript. FG helped to draft the manuscript and involved in revising it critically for important intellectual content. CO participated in acquisition of data. LD participated in the study design and performed the statistical analyses. DC and MG participated in the study design. All authors read and approved the final version of the manuscript.

## Pre-publication history

The pre-publication history for this paper can be accessed here:


